# Influenza Vaccine With Consensus Internal Antigens as Immunogens Provides Cross-Group Protection Against Influenza A Viruses

**DOI:** 10.3389/fmicb.2019.01630

**Published:** 2019-07-16

**Authors:** Xinci Xie, Chen Zhao, Qian He, Tianyi Qiu, Songhua Yuan, Longfei Ding, Lu Liu, Lang Jiang, Jing Wang, Linxia Zhang, Chao Zhang, Xiang Wang, Dongming Zhou, Xiaoyan Zhang, Jianqing Xu

**Affiliations:** ^1^Shanghai Public Health Clinical Center and Institutes of Biomedical Science, Shanghai Medical College, Fudan University, Shanghai, China; ^2^Vaccine Research Center, Key Laboratory of Molecular Virology and Immunology, Institut Pasteur of Shanghai, Chinese Academy of Sciences, Shanghai, China

**Keywords:** universal influenza vaccine, consensus sequence, CD8+ T cell epitope, cross-protection, lung residential T cells

## Abstract

Given that continuing antigenic shift and drift of influenza A viruses result in the escape from previous vaccine-induced immune protection, a universal influenza vaccine has been actively sought. However, there were very few vaccines capable of eliciting cross-group ant-influenza immunity. Here, we designed two novel composite immunogens containing highly conserved T-cell epitopes of six influenza A virus internal antigens, and expressed them in DNA, recombinant adenovirus-based (AdC68) and recombinant vaccinia vectors, respectively, to formulate three vaccine forms. The introduction of the two immunogens via a DNA priming and viral vectored vaccine boosting modality afforded cross-group protection from both PR8 and H7N9 influenza virus challenges in mice. Both respiratory residential and systemic T cells contributed to the protective efficacy. Intranasal but not intramuscular administration of AdC68 based vaccine was capable of raising both T cell subpopulations to confer a full protection from lethal PR8 and H7N9 challenges, and blocking the lymphatic egress of T cells during challenges attenuated the protection. Thus, by targeting highly conserved internal viral epitopes to efficiently generate both respiratory and systemic memory T cells, the sequential vaccination strategy reported here represented a new promising candidate for the development of T-cell based universal influenza vaccines.

## Introduction

Influenza A virus (IAV) has continued to be a major threat to human health (Horimoto and Kawaoka, 2005; Gostin et al., 2014). The need for cross-protective IAV vaccines has arisen in recent years after several global outbreaks of IAV strains such as avian H5N1, swine H1N1, canine H3N2, and avian H7N9 (Gao et al., 2013). However, the licensed influenza vaccines, the majority of which act by inducing antibody against the viral hemagglutinin surface protein, only induce strain-specific immunity and could not provide efficient protection against mismatched epidemic and pandemic influenza variants which have continually emerged by antigenic drift or shift (Hannoun, 2013; Houser and Subbarao, 2015).

Alongside the humoral response, cellular response is the other arm of human immunity battling against influenza infection. A recent study on the victims of 2013 H7N9 outbreak revealed that patients making faster recovery exhibited earlier prominent H7N9-specific CD8+ T-cell responses than those who required longer hospitalization (Wang et al., 2015). There was also evidence supporting the important protective role of CD8+ T-cell response in human adults upon pandemic H1N1 infection (Sridhar et al., 2013). Notably, unlike the surface viral proteins which evolve fast in response to the pressure of human neutralizing antibodies, the internal IAV structural proteins, as exampled by matrix protein (Fan et al., 2004; Valkenburg et al., 2014), nuclear protein (Lamere et al., 2011; Pica and Palese, 2013), and polymerase protein (Cox and Dewhurst, 2015; Uddback et al., 2016) are more conserved and the derived conserved epitopes have shown potential to induce broad-spectrum cellular responses and provide cross-protection (Jaiswal et al., 2013). It’s also worth mentioning that tissue-resident memory CD8 T cells have been reported to be indispensable for cross-protection against different strains of influenza virus (Brown and Kelso, 2009).

A variety of approaches have been attempted to development universal influenza vaccines. The vaccination approaches using attenuated and inactivated influenza viruses have been most popular but the lack of effectiveness and productivity has always been of concern (Holloway et al., 2014). A growing number of novel strategies have been also explored including DNA vaccines, viral vector-based vaccines and combinatorial strategies with DNA vaccine as prime and viral vector-based vaccine or purified protein subunit as boost (Draper and Heeney, 2010), some of which have even been evaluated in clinical trials (Stanekova and Vareckova, 2010). Among all the viral vector-based platforms, adenovirus-based vector holds great promise because of its broad cell tropism, strong genetic stability, high transduction efficiency, and high gene expression. The most common adenovirus vector is human Ad serotype 5 (AdHu5); however, it has already elicited neutralizing antibodies in a large number of people, which greatly limits its clinical application (Wang et al., 2014). In this regard chimpanzee adenovirus serotype 68 (AdC68) has been recently identified as a potential candidate vaccine vector more suitable for human use than AdHu5, owing to its significantly lower seropositivity rate in humans (Zhang et al., 2013; Xing et al., 2016). Another viral vector-based platform of interest is attenuated, replication-competent TIANTAN vaccinia (TTV) virus which has the ability to infect many cell types, and induce both antigen-specific antibody titers and cellular response. The potential of TTV virus for vaccine development is substantiated by the finding that the administration of this virus is safe in human, including people with compromised immune systems (Huang et al., 2007a,b).

In this study, we utilized different vector-based platforms to develop influenza vaccines which are tailored to elicit broad T cell response targeting conserved viral epitopes by expressing conserved sequences of influenza internal proteins. Specifically, we deduced the consensus amino acid sequence of six conserved internal proteins-M1, M2, NP, PA, PB1, and PB2 from approximately 40,000 IAV strains. Consequently, two vaccine sequences were designed according to computation-based prediction of conservative CD8+ T cell epitopes, and used to generate vaccines based on different vector-based platforms, including DNA vaccines, recombinant chimpanzee adenovirus-based vaccines and a recombinant TIANTAN vaccinia vaccine. The immunogenicity and efficacy of these vaccines as well as their combined administration were evaluated in mouse models. The results revealed that both AdC68 vaccine and TTV vaccine, in conjunction with priming of DNA vaccine, were able to elicit significant protective CD8+ T cell response, although the potency and breadth of such responses differs between the two types of vaccine. Further exploration of sequential vaccinations and administration route identified the regimen of DNA priming followed by consecutive boosting with AdC68 vaccine via intranasal route and TTV vaccine via intramuscular route as the most efficient regimen in conferring protection against lethal challenges of H1N1 and H7N9 influenza virus. Together, these data demonstrated DNA prime-novel viral vector boost to deliver conserved CD8+ T cell epitopes of viral proteins as a promising strategy to develop universal influenza vaccines capable of cross-group protection.

## Materials and Methods

### Immunogen Design and Optimization

The protein sequences of approximately 40,000 IAV strains were downloaded from Genbank database, and multiple sequence alignment was conducted using CLUSTAL X program to identify the consensus amino acid at each position. The consensus protein sequence was synthesized by combining individual consensus amino acid; only partial sequences of PA, PB1, and PB2 were encompassed on the basis of CD8+ T cell epitope prediction through online tools (Singh and Raghava, 2003; Moutaftsi et al., 2006). The sequences were optimized with respect to mammalian codon usage.

### Vaccine Construction and Validation

The PAPB1M1 and NPPB2M2 immunogens were cloned into three types of vectors for vaccine construction. For DNA vaccine, pSV1.0 vector was used as the backbone vector. For adenovirus-based vaccine, the immunogen sequences were first cloned into p-Shuttle vector and subsequently subcloned into the E1/E3 deleted AdC68 vector. The resulting vectors, namely AdC68-PAPB1M1 and AdC68-NPPB2M2, were linearized and transfected into HEK293 cells to generate adenoviruses which were purified from the supernatant by CsCl gradient centrifugation followed by determination of virus particle number by UV absorbance. For TTV vaccinia-based vaccines, the immunogen sequences were cloned into pSC65 shuffle vector and then transfected into TK143 cells. The transfected cells were subsequently infected with recombinant wild-type Tiantan virus followed by BrdU screening, yielding recombinant virus which was amplified using Vero cells. For validation of vaccines in immunogen expression, the DNA-based vaccine vectors were transfected into HEK293 cells and the AdC68-based and TTV vaccinia-based virus vaccines were used to infect HEK293 and Vero cells, respectively. The transfected or infected cells were harvested 24 or 48 h late and the immunogens presented in the resulting lysates were detected by immunobloting using anti-M1 monoclonal antibody (abcam, ab22396) or anti-M2 monoclonal antibody (Santa Cruz, sc-52026).

### Mouse Immunization

Six- to eight-week-old female C57BL/6 mice were purchased from the B&K Universal Group Ltd. (Shanghai, China) and housed under specific pathogen-free (SPF) conditions at the animal facilities of Shanghai Public Health Clinical Center, Fudan University (Shanghai, China). For immunization, two doses of pSV1.0-PAPB1M1 (50 μg) and pSV1.0-NPPB2M2 (50 μg) were used as prime and AdC68-PAPB1M1 (5 × 10^10^ vp)/AdC68-NPPB2M2 (5 × 10^10^ vp) or TTV-2a (1 × 10^7^ pfu) was used individually or in sequential combination as boost(s). The mice were immunized following the indicated schedules and route. For sham control, either only the priming vector was substituted by empty vector or both the priming and boosting vectors were replaced with empty vectors. Four weeks after vaccination, mice were sacrificed for immunogenicity evaluation including IFN-γ ElISpot assay and intracellular cytokine staining.

### T-Cell Response Determination

Immunogenicity evaluation was performed 4 weeks after last vaccination. Control or vaccinated mice were sacrificed for isolating splenocytes and bronchoalveolar lavage cells. A total of 2 × 10^5^ isolated cells were plated in triplicate in 96-well plates pre-coated with 5 μg/ml of purified anti-mouse IFN-γ and subsequently stimulated with a peptide specific for one of the six viral immunogens (M2, M1, NP, PA, PB1, and PB2) at a final 5 μg/ml concentration (Table 1). A total of 16 peptides were used with one for M2 and three each for the rest five immunogens. After 24 h stimulation, the cells were washed with deionized water and exposed to 100 μl biotinylated anti-mouse IFN-γ (2 μg/ml) for 2 h at room temperature, followed by extensive washing prior to the addition of 100 μl Streptavidin-HRP. After 1 h incubation at room temperature, the cells were washed and 100 μl of substrate solution was added to develop spots. The reaction was stopped with water and the number of spot-forming cells (SFCs) was determined using an automated ELISPOT software (Saizhi, Beijing, China). For intracellular staining of cytokines, 2 × 10^6^ immune cells were stimulated for 1 h with a peptide pool consisting of equal amount of all the 16 peptides described in Table 1 in the presence of anti-mouse CD107a-PE (BioLegend) antibody, followed by exposure to 1 μl/ml Brefeldin (BD Bioscience) for 6 h. The cells were then washed, and stained with the surface-specific mouse antibodies, LIVE/DEAD-AmyCan, CD3-PerCP-cy5.5, CD8-PB (BioLegend). Cells were subsequently permeabilized using the BD Cytofix/Cytoperm Kit and stained for the intracellular cytokines by FITC anti-mouse IFNγ antibody and PECy7 anti-mouse TNFα antibody (BioLegend). Samples were measured using Fortessa Flow cytometer (BD Bioscience), and the data were analyzed with FlowJo 10.0.6 software (Tree Star).

**TABLE 1:** Influenza A virus-specific peptides employed to stimulate splenocytes for the quantification of T cell responses.

**Protein**	**Amino acid sequence of peptides**
M1	58–66:GILGFVFTL (Halstead et al., 2002) 128–135:MGLIYNRM (Lawrence et al., 2005) 99–107:LYRKLKREI
M2	2–24:SLLTEVETPIRNEWGCRCNDSSD
NP	146–155:ATYQRTRALV (Turner et al., 2004) 366–374:ASNENMDTM (Tussey et al., 1995) 383–391:SRYWAIRTR (Belz et al., 2001)
PB1	703–711:SSYRRVPGI (Chen et al., 2001) 415–424:VSGVNESADM 494–502:DGGPNLYNI
PB2	198–206:ISPLMVAYM (Zhu et al., 2013) 344–352:VLTGNLQTL 544–553:SVLVNTYQWI
PA	509–518:SHLRNDTDVV 515–523:TDVVNFVSM 601–609:VEEGSIGKV

### Influenza A Virus Challenge

Four weeks after vaccination, mice were challenged with either IAV/PR8(H1N1) virus or IAV/Shanghai/4664T/2013(H7N9) virus. The dosage for lethal challenge for both viruses was 500 50% tissue culture-infective dose (TCID50), while a dosage of 100 TCID50 of H7N9 virus was employed for sub-lethal challenge. For assaying the impact of FTY720, experimental groups were split into two halves, one of which was *ad libitum* exposed to drinking water containing 2 μg/ml FTY720 during the whole duration of virus challenge. The body weight and survival rate were daily monitored for 14 days. Lung viral loads were determined on day 5 post infection by quantification of viral RNA: total RNA was extracted from lung tissues and subjected to TaqMan real-time reverse transcription-PCR (RT-PCR) using influenza virus-specific primers for determination of relative levels of viral loads. For normalization, glyceraldehyde phosphate dehydrogenase were used as the reference gene. The used primers were: For H7N9 virus detection, end primer pair as of GAAGAGGCAATGCAAAATAGAATACA and CCCGAAG CTAAACCARAGTAT CA, probe as of CCAGTCAAACTAAG CAGYGGCTACAAA; for PR8 virus detection, end primer pair as of GACCGATCCTGTCACCTCTGA and AGGGCAT TCTGGACAAA GCG TCTA, probe as of TGCAGTCCTC GCTCACTGGGCACG -3′; for GAPDH reference detection, end primer pair as of CAATGTGTCCGTCGTGGA TCT and GTCCTCAGTGTAGCCCAAGATG, probe as of CGTGCC GCCTGGAGAA ACCTGCC. The animal studies were carried out in accordance with the “Guide for the Care and Use of Laboratory Animals of the Institute of Laboratory Animal Science (est. 2006).” Mice that lost over 30% of their initial body weight were scored dead and humanely euthanized. All other mice were humanely euthanized after 14-day observation period. The H7N9 virus-related experiments were conducted in a biosafety level 3 laboratory following protocols approved by the Institutional Biosafety Committee at Shanghai Public Health Clinical Center.

### Statistical Analysis

All statistical analyses were performed using GraphPad Prism 6.0 (GraphPad Software, Inc.). Mantel-Cox log rank test and two-way ANOVA test were applied to evaluate difference in survival and weight loss, respectively. In other cases, *t*-test was used. Significant difference was defined as *p* < 0.05.

## Results

### Construction of Influenza Internal Gene Based Vaccines

**FIGURE 1:**
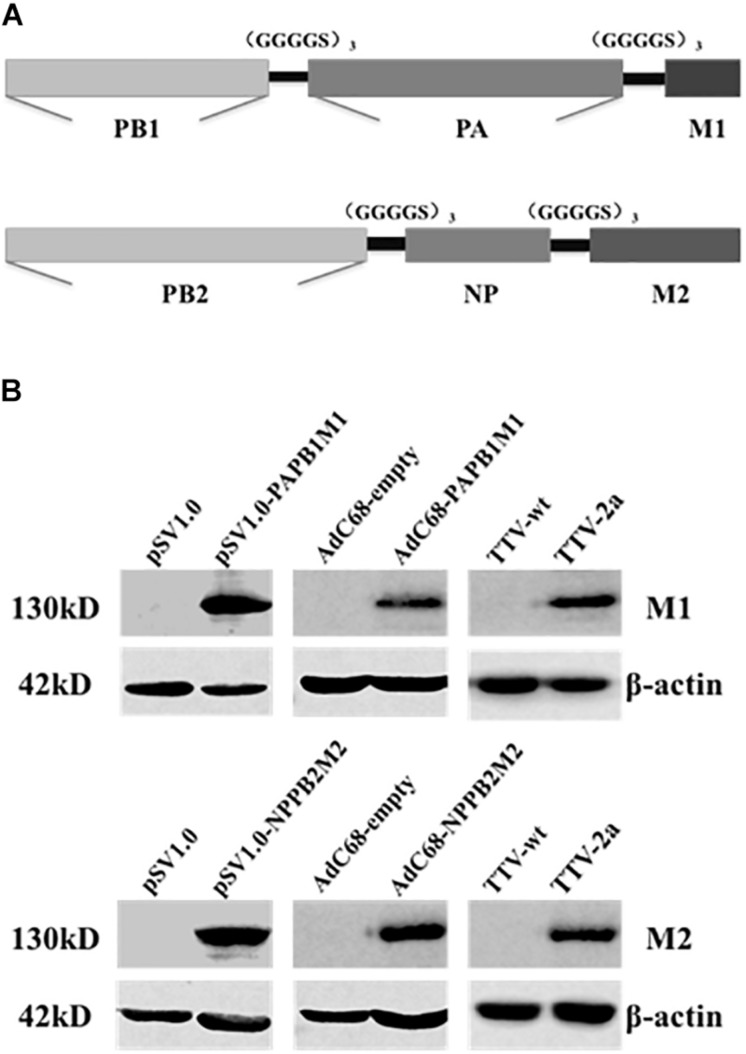
Immunogen design and expression through three different vaccine platforms. **(A)** Schematic diagram of two synthetic immunogens, PB1PAM1 and PB2NPM2, which were designed on the basis of amino acid conservation and CD8+ T cell epitope prediction of influenza M1, M2, NP, PA, PB1, and PB2 sequences. **(B)** Validation of vaccine-generated PAPB1M1 and PB2NPM2 protein expression in cultured cells. HEK293 cells were used for the transfection of pSV1.0-based vectors or the infection with AdC68-based vectors, while Vero cells were used for TTV infections. The resulting cell lysates were resolved by denaturing electrophoresis followed by western blotting using antibodies against influenza M1 or M2 protein, or anti-β-actin antibodies as internal control. The cell lysates yielded from transfection or infection of corresponding empty vector were used as negative controls.

As the first step to develop new cross-protective IAV vaccine, we sought to identify new immunogens that have a broad coverage of conserved CD8+ T cell epitopes of IAV antigens. To this end, we deduced the consensus amino acid sequences of influenza M1, M2, NP, PA, PB1, and PB2 proteins from approximately 40,000 IAV strains available in Genebank database. To be more efficient in immunogen design, we only included partial sequences of PA, PB1, and PB2 enriched with CD8+ T cell epitopes as predicted by online tools (Singh and Raghava, 2003; Moutaftsi et al., 2006). Consequently, we generated two immunogen sequences, denoted as PAPB1M1 and PB2NPM2, whose protein composition were schematically illustrated in Figure 1A and amino sequences were included in the Supplementary Material.

We thus constructed vaccines to express the two immunogens in three platforms including DNA vector, E1/E3-deleted replication-deficient chimpanzee Adenovirus (AdC68), and recombinant Tiantan vaccinia virus (TTV). For the first two platforms, two immunogens were expressed separately, resulting in two DNA-based vaccines (pSV1.0-PAPB1M1 and pSV1.0-PB2NPM2) and two AdC68-based vaccines (AdC68-PAPB1M1 and AdC68-PB2NPM2); for TTV platform, two immunogens were expressed from a single vaccinia vaccine, namely TTV-2a. The resulting vaccines were introduced into cultured cells by either transfection or infection, and their expressions of encoded immunogens in the cells were validated by immunoblotting using antibodies specific for IAV M1 or M2 protein (Figure 1B). Thus, all three platforms were capable of expressing PAPB1M1 and PB2NPM2 immunogens efficiently.

### DNA Prime and Viral Vectored Boost Efficiently Mounted Influenza-Specific CD8+ T Cell Responses

**FIGURE 2:**
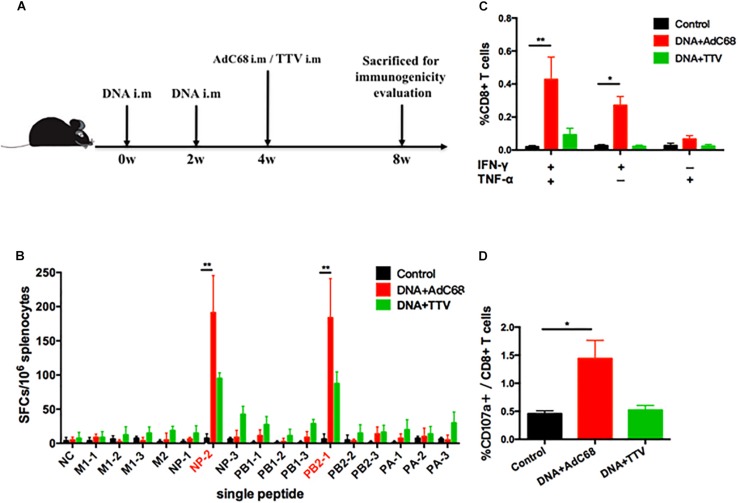
Influenza-specific T-cell immune responses raised by DNA prime-viral vectored vaccine boost. **(A)** Schematic representation of immunization regimens. **(B–D)** Cellular responses elicited in vaccinated mice. Splenocytes of vaccinated C57 mice were isolated 4 weeks after vaccination and subjected to both IFN-γ ELISpot assay **(B)** and intracellular cytokine staining assay **(C,D)**. The IFN-γ ELISpot assays were performed in response to stimulation with a single IAV virus-specific peptide and described as numbers of spot forming cells (SFCs) per 10^6^ splenocytes. A total of 16 peptides were tested (Table 1). The intracellular cytokine staining assays were performed upon stimulation with a peptide pool composed of all the 16 peptides, and represented as the percentage of CD8+ T cells that were single positive or double positive for IFN-γ, TNF-α, and CD107a expression. All experiments were carried out in triplicate; the error bars are represented as SDs. ^∗∗^*p* < 0.01; ^*^p < 0.05, *t*-test.

We next examined the capability of our newly developed vaccines in a DNA prime-viral vectored boost modality to induce influenza-specific cellular immune responses in mice. The mice were divided into three groups: the control group was immunized intramuscularly with two doses of control vector pSV1.0 (100 ug) and one dose of control vector AdC68-empty (1 × 10^11^ vp); the two experimental groups, namely DNA+AdC68 and DNA+TTV group, were immunized intramuscularly twice with pSV1.0-PAPB1M1 (50 μg)/pSV1.0-NPPB2M2 (50 μg), followed by intramuscularly boosting with AdC68-PAPB1M1 (5 × 10^10^ vp)/AdC68-NPPB2M2 (5 × 10^10^ vp) or TTV-2a (1 × 10^7^ pfu), respectively (Figure 2A). Four weeks after vaccination, three mice in each group were euthanized and splenocytes were isolated for measuring influenza-specific CD8+ T cell response by IFN-γ ELISpot assay (Figure 2B) and intracellular cytokine staining (ICS) assay (Figures 2C,D).

The data showed that, among the 16 influenza-specific epitope peptides examined (Table 1), NP-2 and PB2-1 peptides were dominant in inducing IFN-γ-producing immune cells for both experimental groups. However, DNA+AdC68 group exhibited a noticeably higher response to either of these two peptides than DNA+TTV group (Figure 2B). In addition, significantly more IFN-γ+/TNF-α+, IFN-γ+ cells, and CD107a+ cells appeared in DNA+AdC68 group as compared to DNA+TTV group after stimulation with a peptide pool composed of all the 16 peptides (Figures 2C,D). In contrast, DNA+TTV group exhibited a broader cellular response, as was indicated by more peptides, e.g., M2, NP-3, PB1-1, PB1-3, PA-1, and PA-3, eliciting modest but detectable IFN-γ induction in immune cells from this group (Figure 2B). Thus, DNA+AdC68 regimen was more effective in raising immunodominant T-cell responses whereas DNA+TTV regimen appeared to perform better in eliciting sub-immunodominant T-cell responses. We also compared the serum levels of anti-M2 and anti-NP IgG between immunized and sham groups. The results showed that the two immunogens were ineffective in raising significant antibody response to either of the two viral proteins, which were also true for other vaccination regimens we later examined (Supplementary Appendix 1).

### DNA Prime/Viral Vectored Boost Regimens Afforded Protection Against Heterologous Influenza Virus Challenges in Murine Model

To determine the protective efficacy of the DNA prime/viral vectored boost regimens, we utilized a murine model of influenza challenge. Mice were immunized following schedule as described in Figure 2A and, split into two groups, which were respectively challenged with either A/PR8(H1N1) (a group 1 IAV) or A/Shanghai/4664T/2013(H7N9) (a group 2 IAV) 4 weeks later.

**FIGURE 3:**
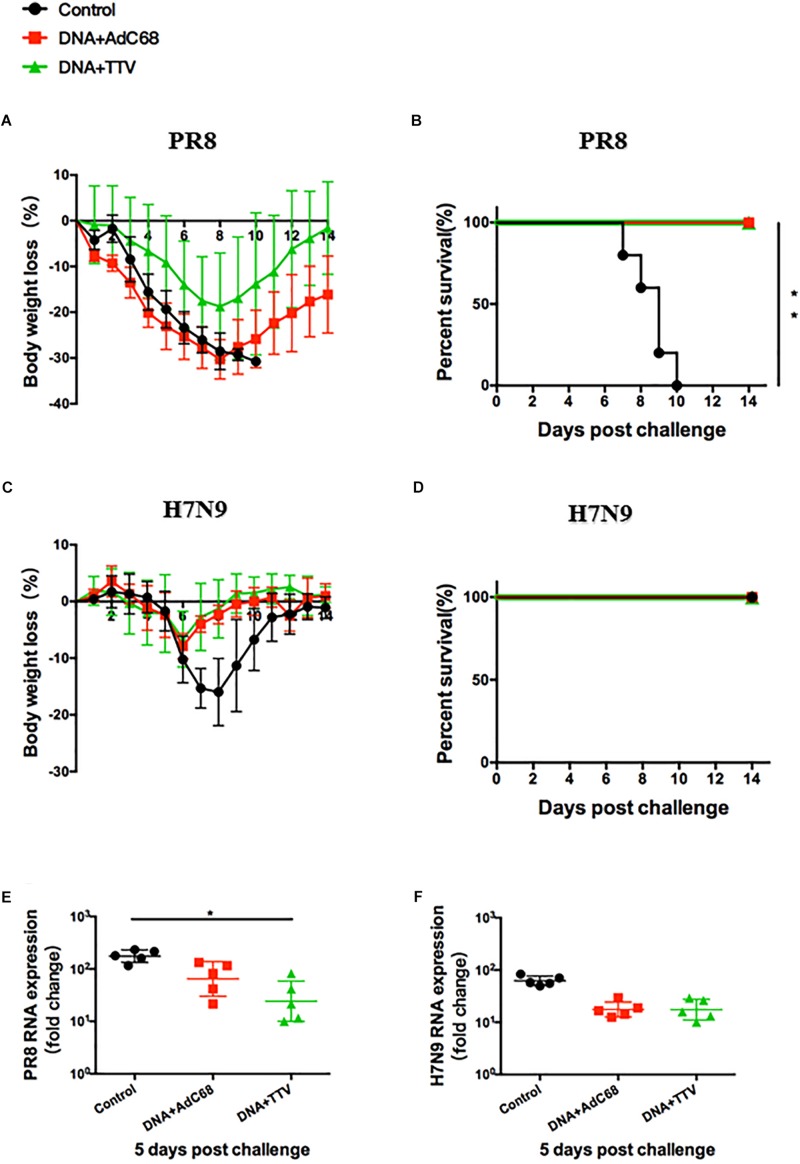
DNA prime-viral vectored vaccine boost strategies conferred protection against PR8/(H1N1) and H7N9 viruses in vaccinated mice. Mice were immunized following schedule outlined in Figure 2A, and 4 weeks later challenged with either 500 50% tissue culture-infective dose (TCID50) of A/PR8(H1N1) or 100 TCID50 A/Shanghai/4664T/2013(H7N9) influenza virus. After infection, mice (*n* = 5 in each group) were monitored daily for body weight **(A,C)** or survival **(B,D)**. Five mice in each group were sacrificed on day 5 post infection to isolate lung tissues for RNA extraction, followed by RT-PCR quantification of viral RNA to determine the relative viral loads **(E,F)**. The error bars represent the SDs. Two-way ANOVA test, Mantel–Cox log rank test and *t*-test were used to determine difference in weight loss, lethality, and viral load, respectively. ^∗∗^*p* < 0.01; ^*^p < 0.05.

After a fatal challenge with A/PR8 (H1N1) virus, mice in sham control rapidly and continuously lost their weights (Figure 3A). Consequently, they went to death between day 7 and 12 (Figure 3B). In contrast, although the body weights of the two vaccinated groups also experienced an initial decrease, they reached a nadir on day 8 and then rebound afterward (Figure 3A). Accordingly, all the mice survived (Figure 3B). Furthermore, vaccinated mice showed reduced lung viral loads as compared to control group (Figure 3E). Interestingly, DNA+TTV group underwent a slower and less decrease in their body weights in comparison with DNA-AdC68 group (Figure 3A), which was in line with slightly lower viral loads (Figure 3E), suggesting that DNA+TTV regimen may provide a better protection. Hence, our newly designed T-cell vaccine is capable of affording efficient protection against fatal PR8 challenge.

In the case of H7N9 challenge, both regimens were found to be ineffective in protection from a lethal infection (data not shown). However, they did show protective effects against a non-lethal challenge (Figure 3D), as indicated by less initial loss and earlier recovery of body weights in comparison to control group (*p* < 0.05 during the period of day 6 to day 10 after infection) (Figure 3C). This was in agreement with discernible, although not statistically significant, inhibition of viral replication in lung (Figure 3F). Thus both DNA+AdC68 and DNA+TTV regimens were able to mount some protection from H7N9 infection in murine models.

### Intranasal Administration of Adenoviral Vectored Vaccine Elicits Vigorous Respiratory Residential T-Cell Responses in a Combinatory Regimen

**FIGURE 4:**
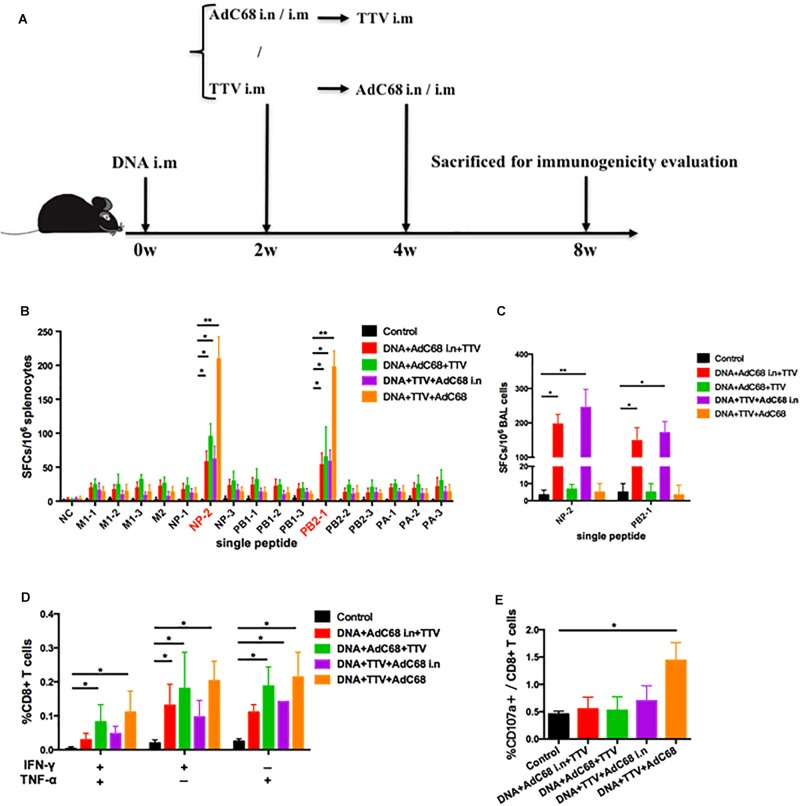
Intranasal administration of AdC68 elicits significantly more potent respiratory residential and less systemic memory T cell responses. **(A)** Schematic illustration of immunization regimens. Control group were immunized with one dose of control pSV1.0 vector (100 ug) via intramuscular (i.m) route and two doses of control AdC68 empty vector (1 × 10^11^ vp) separately via intramuscular (i.m) and intranasal (i.n.) route. Experimental groups were sequentially immunized with DNA, AdC68, and TTV in the indicated order, the intramuscular route was treated as default to be left undenoted whereas the intranasal administration was labeled as i.n. Splenocytes and bronchoalveolar lavage (BAL) were isolated 4 weeks after vaccination for measurement of influenza-specific immune responses. **(B)** IFNγ ELISpot assay of splenocytes in response to stimulation with a single indicated influenza-specific epitope peptide. **(C)** IFNγ ELISpot assay of BAL in response to stimulation with NP-1 and PB2-1 peptides. **(D,E)** Intracellular cytokine staining assay to determine the percentage of CD8+ splenocytes secreting IFN-γ, TNF-α, or both, and CD107a-positive cells after stimulation with the peptide pool. All determinations were carried out in triplicate and the error bars represent the SDs. ^∗∗^*p* < 0.01; ^*^p < 0.05, *t*-test.

To further optimize our vaccination strategy to improve cross-protection, we evaluated the effect of second boost as well as administration route on the vaccine-induced immune responses. Specifically, AdC68 was either intranasally or intramuscularly administered before or after TTV intramuscular inoculation in a combinatorial regimen with a DNA intramuscular priming (Figure 4A). The IFN-γ ELISpot assay of splenocytes isolated from vaccinated mice showed that, among the four tested groups, DNA+TTV+AdC68 group mounted significantly higher systemic cellular responses to immunodominant NP-2 and PB2-1 epitope peptides than the other three groups (Figure 4B). In contrast, the regimens of DNA+AdC68+TTV and DNA+AdC68 i.n.+TTV raised more potent T-cell immune responses against subdominant epitopes than those of the other two regimens (Figure 4B). We further analyzed the T-cell functionalities by measuring the production of intracellular cytokine after stimulation with the peptide pool. As shown in Figure 4D, the intramuscular administration of AdC68 induced more influenza-specific IFN-γ+, TNF-α+ and IFN-γ+TNF-α+ double positive T cells than their intranasal counterparts. Among all the four groups, DNA+TTV+AdC68 group exhibited the highest induction of CD107a+ cells (*p* = 0.049) (Figure 4E).

Given the potential important role that lung-resident T cells play in anti-influenza immunity, we extended our IFN-γ ELISpot analysis to bronchoalveolar lavage (BAL) lymphocytes. Strikingly, only the two intranasal groups showed robust BAL T-cell immune response upon stimulation with NP-2 and PB2-1 epitope peptides (Figure 4C). Thus, intranasal administration of AdC68 appeared to have a pronounced advantage over intramuscular administration in the induction of respiratory resident T cells.

### Intranasal Administration of AdC68-Based Vaccine Afforded a Better Protection From Lethal Influenza Challenge in Mice

**FIGURE 5:**
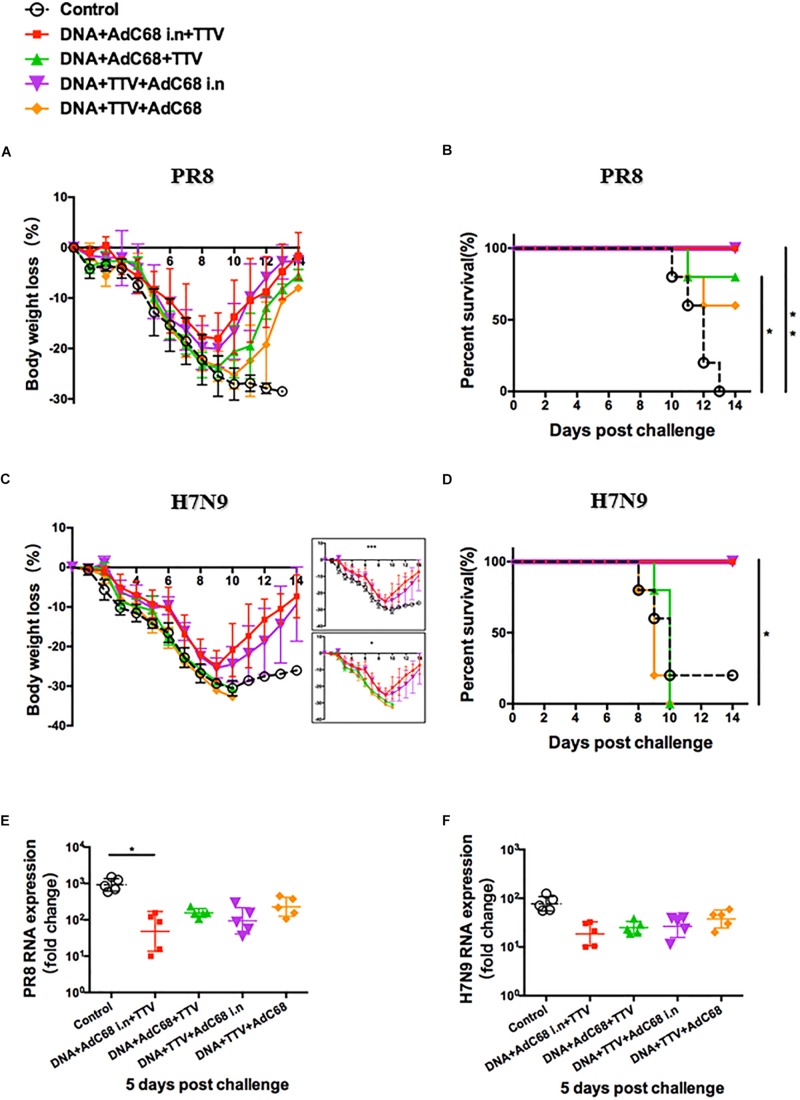
Intranasal administration of AdC68 afforded a better protection from lethal challenge of influenza. Mice were subjected to different combinatorial immunizations following schemes outlined in Figure 4A, and 4 weeks later challenged with lethal doses of PR8 or H7N9 IAV virus. Shown are: **(A,C)** Body weight curves of virus-challenged mice. **(B,D)** Survival curve of virus-infected mice. **(E,F)** Relative lung viral loads at day 5 after virus challenge as measured by RT-PCR quantifications of influenza-specific RNA. The error bars represent the SDs. Two-way ANOVA test, Mantel–Cox log rank test and *t*-test were used to determine difference in weight loss, lethality, and viral load, respectively. ^∗∗^*p* < 0.01; ^*^p < 0.05; *n* = 5. Weight loss in the two intranasal immunization groups were significantly different from both the two non-intranasal immunization groups and control group with *p* < 0.001 and *p* < 0.05, respectively.

We next evaluated the *in vivo* protective efficacy of prime-boost-boost immunization regimens against PR8 and H7N9 challenges. In response to lethal PR8 challenge, the two AdC68 intranasal immunization groups experienced similar weight losses that were less severe than the two intramuscular immunization groups (Figure 5A). This was marked by a slower initial loss and an earlier rebound of weights on day 9 as compared to day 10 in the later groups, resulting in a higher nadir weight before rebounding. Consequently, all the mice from the two intranasal immunization groups survived whereas the two intramuscular immunization groups only attained 60–80% survival rate. In contrast, all the mice from the sham control group died (Figure 5B). We further found that intranasal administration appeared to result in more reduced lung vial loads, which was especially obvious with the DNA+Adc68 i.n.+TTV group (*p* = 0.046) (Figure 5E). Thus, although all the four combinatorial immunization regimens were able to confer protection against PR8 infection, the two regimens with intranasal AdC68 vaccination were more effective.

Our studies on lethal H7N9 challenge models further supported that the intranasal route was superior to intramuscular route in protection. In particular, both DNA+Adc68 i.n.+TTV and DNA+TTV+Adc68 i.n. groups exhibited a body weight loss curve similar to that observed in PR8 challenge with the body weight reaching a nadir at day 9 and then rebounding afterward, differing significantly from DNA+Adc68+TTV, DNA+TTV+Adc68 and control groups which all suffered more rapidly and continuous weight loss without recovery (Figure 5C). Consequently, the two intranasal immunization groups all survived whereas the majority of the two intramuscular immunization groups died (Figure 5D). Interestingly, the measured lung viral load at day 5 after challenge revealed only modest advantage of intranasal vaccination over intramuscular vaccination (Figure 5F). Collectively, these data demonstrated that intranasal route is an optimal route for Adc68-based vaccine to achieve cross-group influenza protection in prime-boost-boost modality.

### Both Respiratory Residential and Systemic Memory T Cells Are Essential for Fully Protection

**FIGURE 6:**
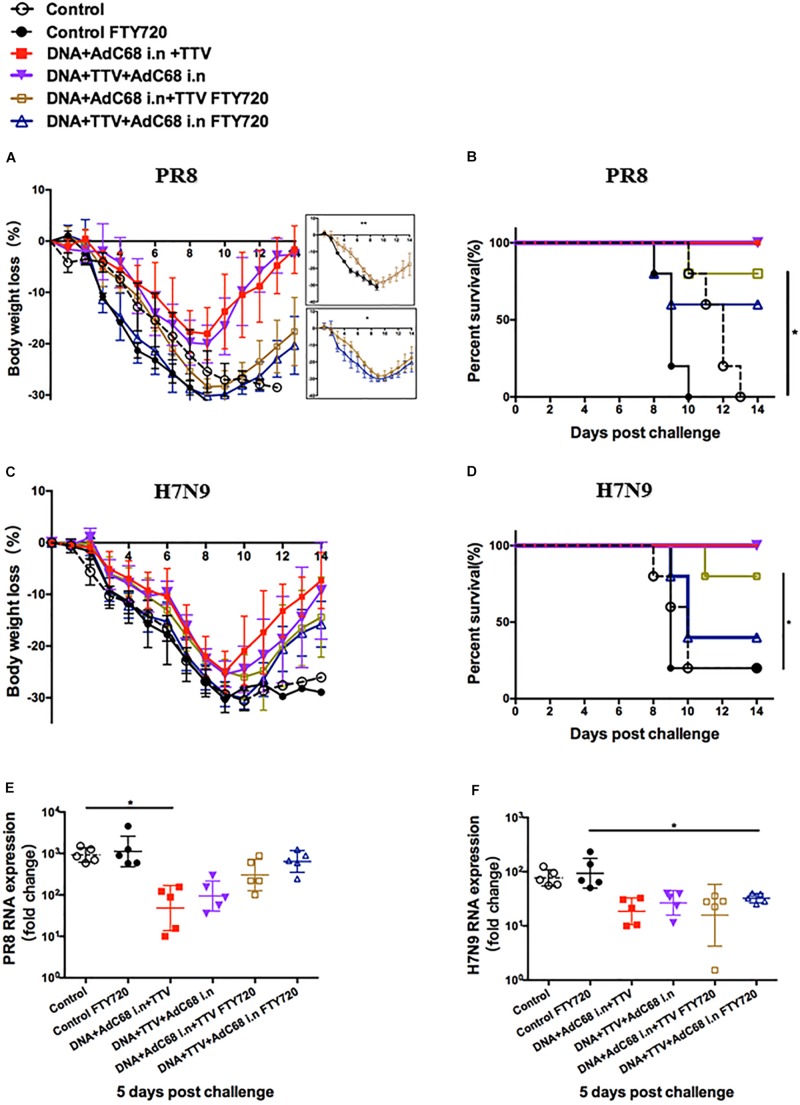
Both respiratory residential and systemic T cells are essential for fully protection. The sequential immunizations and virus challenges of vaccinated mice were essentially performed as described in Figure 5A, except that, where indicated, the mice were exposed *ad libitum* to drinking water containing 2 μg/ml of dissolved FTY720 during challenge. Shown are: **(A,C)** Body weight curve of virus-challenged mice. **(B,D)** Survival curve of virus-challenged mice. **(E,F)** Relative lung viral loads in infected mice at day 5 after virus challenge as measured by RT-PCR quantifications of influenza-specific RNA. The error bars represent the SDs. Two-way ANOVA test, Mantel–Cox log rank test and *t*-test were used to determine difference in weight loss, lethality, and viral load, respectively. ^∗∗^*p* < 0.01; ^*^p < 0.05; *n* = 5.

Our above results indicated that DNA+AdC68 i.n.+TV and DNA+TTV+Adc68 i.n. regimens were capable of raising both respiratory residential and systemic memory T cells. To dissect the contributions of these two T cell subpopulations to the protective immunity, we took advantage of Fingolimod (FTY720), an immunosuppressant which prevents T cell egress from lymph nodes or spleen by antagonizing sphingosine-1-phosphate while unaffects the residential memory T cells (Masopust et al., 2010). For the sake of reducing animal usage, the experiments presented in Figures 5, 6 were performed in parallel, sharing the sham control and the two intranasal groups without FTY720 treatment.

When exposed to FTY720 during lethal PR8 challenge, both intranasal groups were significantly less protected, showing more rapid body weight loss and much delayed regain of body weight (Figure 6A). Consequently, the survival rate dropped from 100 to 80% and 60% for DNA+AdC68 i.n +TTV group and DNA+TTV+AdC68 i.n., respectively (Figure 6B), concomitant with less suppression of viral replication (Figure 6E). Similar impact of FTY720 treatment was also observed in protection against H7N9 challenge, despite that DNA+TTV+AdC68 i.n. group suffered even a bigger drop in survival (Figures 6C,D,F). Taken together, these data suggest that the protection conferred by our intranasal immunization strategy were attributed to the action of both respiratory residential and systemic T cells.

## Discussion

Current influenza vaccines function by raising humoral immunity against strain-specific HA and NA glycoproteins. Consequently, they failed to confer cross-protection in human and must be re-formulated every year to match the circulating influenza strains (Steel et al., 2010). There has been growing urgency to develop universal IAV vaccines that are capable of affording cross-protection (Grant et al., 2013). The development of cross-reactive influenza vaccines has been explored on both the humoral arm of the immune response directing against the conserved HA stem or M2e, and its cellular arm targeting the internal viral proteins which are much more conserved than surface viral glycoproteins (Saletti et al., 2018). The potential of T-cell based vaccine was further supported by our recent studies of H7N9-infected patients revealing the pivotal role of an effective and timely CD8+ T cell response in overcoming H7N9 infection in human (Wang et al., 2015). Here, we presented new vaccination strategies focusing on the effective delivery of cross-conserved influenza-specific epitopes to induce broad spectrum T-cell response.

We would expect that our newly designed PAPB1M1 and PB2NPM2 immunogens together should provide a close to full coverage of conserved T-cell epitopes internally on IAV. Importantly, the co-introduction of the two immunogens in a modality of DNA prime followed by AdC68 or TTV viral vectored boost elicited not only strong T cell response to immunodominant epitopes but also discernible T cell response to sub-immunodominant epitopes, which was more evident when TTV served as the boost. In current study, we have not yet dissected the respective protective contributions of immune responses to the two immunodominant epitopes, NP-2 and PB2-1, and the subdominant epitopes. This will be only accomplished by future examination of the protective efficacy of mutated immunogens in which NP-2 and PB2-1 epitopes are eliminated in comparison to that of the original immunogens in mouse infection model. Even if the subdominant epitopes were found to be insufficient for raising effective protection against influenza challenge in vaccinated mice, their contribution in broadening the T cell repertoire may be important for a vaccine being effective in a human setting. One of the major challenges in the development of T cell-based universal influenza vaccine is that the T cell response to the same influenza infection or vaccine might vary considerably between individuals, primarily owing to the possession of diverse HLA alleles that restricted the number of viral peptides displayed to T cells for recognition (Clemens et al., 2018). With HLA restriction, an influenza vaccine incorporating a multitude of conserved viral epitopes would more likely provide better population coverage than that concentrated on specific conserved epitopes. Thus, the new vaccines we engineered have the potential to overcome the HLA challenge in human settings, fitting one important criterion of universal influenza vaccines.

Our construction of three types of vaccines to express the PAPB1M1 and PB2NPM2 immunogens enabled us to test a sequential immunization strategy combining the three vaccines. It is a rare opportunity as, for viral vectored vaccine, the second boost with the same vaccine would usually not be beneficial due to the pre-existing vector immunity raised by the first boost. The sequential use of different viral platform-based boost also allows the integration of individual advantages of different viral vectored vaccine to induce potent broad-spectrum T-cell immunity.

In light of recent findings that influenza-specific lung resident T cells is critical for anti-influenza immunity (Wu et al., 2014; Zens et al., 2016), we utilized either intramuscular or intranasal route for administering AdC68 based vaccine and analyzed the impact on induction of memory T cells alongside protective efficacy. Our results demonstrated that, although intramuscular immunization was more effective in triggering systemic CD8+ T cell response, only intranasal immunization was capable of evoking lung-resident CD8+ T cells. Consequently, full protection from lethal PR8 and H7N9 challenges was only observed in intranasal immunization groups. One interesting observation was that the level of lung viral load at early phase of infection was marginally correlated with the protective efficacy in terms of weight loss and survival rate. This might be explained by the polyfunctionality of tissue-residing T cells, engaged in duties other than directly clearing virus-infected cells to provide protection, e.g., secreting cytokines to create a virus-hostile local environment, and/or facilitating recruitment of circulating immune cells into lesioned tissues (Schenkel et al., 2013; Ariotti et al., 2014).

Although intranasal administration of adenovirus vectors has been extensively shown to induce potent immune responses in mice, its use in primates has been much less documented in literature precedence. Given the large difference in oropharyngeal immune system between mice and primates, there is concern whether our promising results in mice can be fully translated to humans. However, a recent study reported intranasal application of adenovirus vector being part of a vector prime-protein boost vaccination modality to afford effective protection in African green monkey model against respiratory syncytial virus (RSV) infection (Eyles et al., 2013). Nevertheless, future studies in non-human primates will be needed to justify our notion that, for T-cell based adenovirus vaccine, intranasal route can achieve better efficacy than intramuscular route via establishing lung-resident CD8 memory cells.

Finally, we discovered that systemic T cell response also mediate the protection afforded by our new intranasal immunization strategy. FTY720 treatment, which prevents homing of circulated T cells to periphery tissues, significantly reduced the protective efficacy. Interestingly, a number of recent studies revealed that circulating T cells can be recruited into lung tissues to convert into residing cells (Slutter et al., 2017; Pizzolla et al., 2018). Thus, systemic and respiratory residential T cell might not act independently, but rather orchestrate together in mounting an effective protection against influenza infection.

As compared to other current available vaccination approaches, our new approach is advantageous in expanding the breadth of induced immune response. According to a position statement regarding universal influenza vaccine recently published by National Institute of Allergy and Infectious Diseases (NIAID), one critical qualification element of a universal influenza virus vaccine is to be effective against both group 1 and group 2 influenza A viruses. Most if not all of the current available vaccination approaches will not meet the criterion. For example, one most recent preclinical study identified live attenuated influenza vaccine (LAIV) in combination with sequential immunization strategy as the most promising vaccination regimen to confer broad protection against group 1 influenza viruses (Liu et al., 2019). However, it is uncertain whether the same approach can achieve similar protective efficacy against group 2 influenza viruses, and if yes whether it is feasible to design right immunogens and regimen for attaining effective cross-group protection. The efficacy of LAIV as well as other vaccines lies in their ability to raise potent influenza-specific antibody response, especially against surface HA protein. Such antibody response was missed in the immunity raised by our new vaccine, possibly accounting for less inhibition of viral replication as compared to other vaccines shown in previous studies. How to modify our strategy to engage the B cell response will await further research. Nevertheless, starting with a demonstration of its ability to confer cross-group protection against lethal IAV challenges in vaccinated mice, the novel vaccine described in this study might open new avenue to tackle the challenge of universal influenza vaccine.

## Data Availability

All datasets generated for this study are included in the manuscript and/or the Supplementary Files.

## Ethics Statement

This study was carried out in accordance with the “Guide for the Care and Use of Laboratory Animals” of the Institute of Laboratory Animal Sciences (est. 2006). The protocol was approved by the Institutional Biosafety Committee at Shanghai Public Health Clinical Center.

## Author Contributions

JX, XZ, and DZ conceived, designed, and supervised the study. XX performed most of the experiments, analyzed the data, and wrote the original draft. CZ (2nd Author) analyzed the data. TQ helped design the immunogen sequences. QH, SY, LD, LL, LJ, JW, LZ, CZ (11th Author), and XW conducted some experiments. CZ (2nd Author), JX, XZ, and DZ edited the manuscript. All authors reviewed and approved the manuscript.

## Conflict of Interest Statement

Shanghai Public Health Clinical Center has filed a patent application with PCT Application No. PCT/CN2018/105020, which covers the PAPB1M1 and PB2NPM2 immunogens and the derived influenza vaccines. XX, XZ, and JX have been listed as inventors of PCT/CN2018/105020.

The remaining authors declare that the research was conducted in the absence of any commercial or financial relationships that could be construed as a potential conflict of interest.
