# Supplementary material for: Influenza Vaccine With Consensus Internal Antigens as Immunogens Provides Cross-Group Protection Against Influenza A Viruses
Source: Front Microbiol. 2019 Jul 16;10:1630. doi: 10.3389/fmicb.2019.01630 (PMC6647892; doi:10.3389/fmicb.2019.01630)
Supplement: Supplementary file 1 [file Table_1.DOCX]

**Supplementary Material**


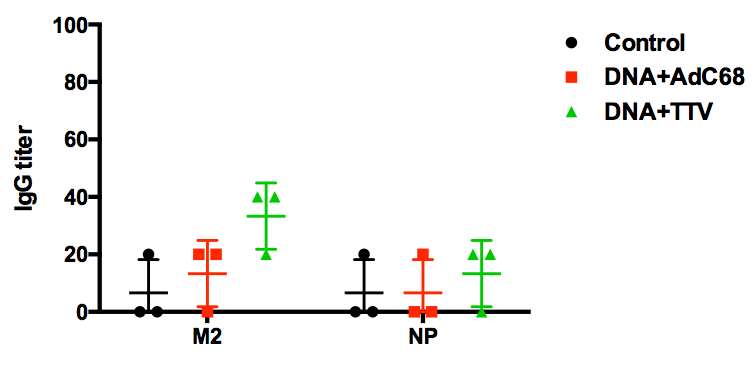

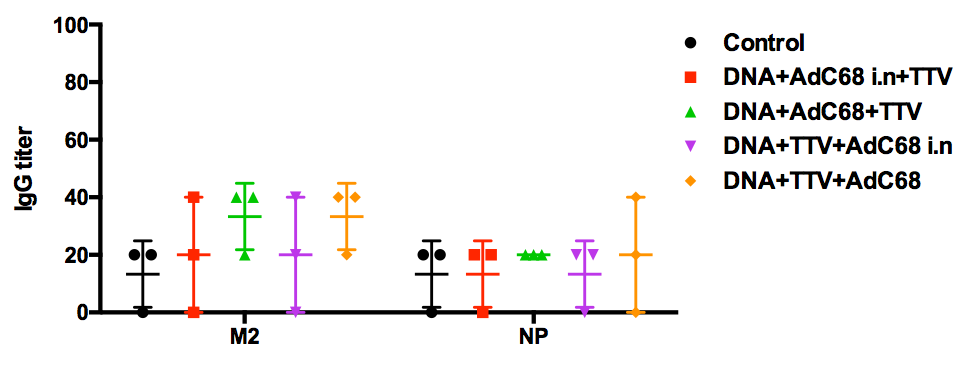


**Appendix 1. Evaluation of influenza-specific antibody response in immunized mice and control mice.** Mice were vaccinated following the indicated modality, and sera were collected at 4 weeks post-vaccination for measurement of anti-M2 and anti-NP IgG titers using ELISA. The error bars represent the SDs. No significant differences were detected between all immunization groups and control groups in terms of IgG level. Upper panel: IgG titers in the prime-boost groups vs control group; lower panel: IgG titers in the primer-boost-boost groups vs control group.

**Appendix 2: Amino acid sequences of the two new T-cell based pan-influenza A immunogens designed and tested in the study**

**Immunogen #1. PAPB1M1 immunogen**

Met Glu Arg Ile Lys Glu Leu Arg Asn Leu Met Ser Gln Ser Arg Thr

1 5 10 15

Arg Glu Ile Leu Thr Lys Thr Thr Val Asp His Met Ala Ile Ile Lys

20 25 30

Lys Tyr Thr Ser Gly Lys Trp Met Met Ala Met Lys Tyr Pro Ile Thr

35 40 45

Ala Asp Lys Arg Ile Thr Glu Met Ile Pro Glu Arg Asn Glu Gln Gly

50 55 60

Gln Thr Leu Trp Ser Lys Met Asn Asp Ala Gly Ser Asp Arg Val Met

65 70 75 80

Val Ser Pro Leu Ala Val Thr Trp Trp Asn Arg Asn Gly Pro Val Thr

85 90 95

Ser Thr Val His Tyr Pro Lys Val Tyr Lys Thr Tyr Phe Glu Lys Val

100 105 110

Glu Arg Leu Lys His Gly Thr Phe Gly Pro Val His Phe Arg Asn Gln

115 120 125

Val Lys Gln Leu Thr Ile Thr Lys Glu Lys Lys Glu Glu Leu Gln Asp

130 135 140

Cys Lys Ile Ser Pro Leu Met Val Ala Tyr Met Leu Glu Arg Glu Leu

145 150 155 160

Val Arg Lys Thr Arg Phe Leu Pro Val Ala Gly Gly Thr Ser Ser Val

165 170 175

Tyr Ile Glu Val Leu Ile Val Arg Arg Ala Ala Val Ser Ala Asp Pro

180 185 190

Leu Ala Ser Leu Leu Glu Met Cys His Ser Gly Leu Arg Ile Ser Ser

195 200 205

Ser Phe Ser Phe Gly Gly Phe Thr Phe Lys Arg Thr Ser Gly Ser Ser

210 215 220

Val Lys Lys Glu Glu Glu Val Leu Thr Gly Asn Leu Gln Thr Leu Lys

225 230 235 240

Ile Arg Ile Val Ser Gly Arg Asp Glu Gln Ser Ile Ala Glu Ala Ile

245 250 255

Ile Val Ala Met Val Phe Ser Pro Met His Gln Leu Leu Arg His Phe

260 265 270

Gln Lys Asp Ala Lys Val Leu Phe Gln Asn Trp Gly Ile Glu His Ile

275 280 285

Asp Asn Val Met Gly Met Ile Gly Ile Leu Pro Asp Met Thr Pro Ser

290 295 300

Thr Glu Met Ser Val Ser Ile Asp Arg Phe Leu Arg Val Arg Asp Gln

305 310 315 320

Arg Gly Asn Val Leu Leu Ser Pro Glu Glu Val Ser Glu Thr Gln Gly

325 330 335

Thr Glu Lys Leu Thr Ile Thr Tyr Ser Ser Ser Met Met Trp Glu Ile

340 345 350

Asn Gly Pro Glu Ser Val Leu Val Asn Thr Tyr Gln Trp Ile Ile Arg

355 360 365

Asn Trp Glu Ala Val Lys Ile Gln Trp Ser Gln Asn Pro Thr Met Leu

370 375 380

Tyr Asn Lys Met Glu Phe Glu Pro Phe Gln Ser Leu Val Pro Lys Ala

385 390 395 400

Ile Arg Ser Gln Tyr Ser Gly Phe Val Arg Thr Leu Phe Gln Gln Met

405 410 415

Arg Asp Val Leu Gly Thr Phe Asp Thr Thr Gln Ile Ile Lys Leu Leu

420 425 430

Pro Phe Ala Ala Ala Pro Pro Lys Gln Ser Arg Met Gln Phe Ser Ser

435 440 445

Leu Thr Val Asn Val Arg Gly Ser Gly Met Arg Ile Leu Val Arg Gly

450 455 460

Asn Ser Pro Val Phe Asn Tyr Asn Lys Thr Thr Lys Arg Leu Thr Val

465 470 475 480

Leu Gly Lys Asp Ala Gly Thr Leu Thr Glu Asp Pro Asp Glu Gly Thr

485 490 495

Ser Gly Val Glu Ser Ala Val Leu Arg Gly Phe Leu Ile Leu Gly Lys

500 505 510

Glu Asp Arg Arg Tyr Gly Pro Ala Leu Ser Ile Asn Glu Leu Ser Val

515 520 525

Met Lys Arg Lys Arg Asp Ser Ser Ile Leu Thr Asp Ser Gln Thr Ala

530 535 540

Thr Lys Arg Ile Arg Met Ala Ile Asn Gly Ser Gly Gly Ser Gly Met

545 550 555 560

Ala Ser Gln Gly Thr Lys Arg Ser Tyr Glu Gln Met Glu Thr Asp Gly

565 570 575

Glu Arg Gln Asn Ala Thr Glu Ile Arg Ala Ser Val Gly Arg Met Ile

580 585 590

Gly Gly Ile Gly Arg Phe Tyr Ile Gln Met Cys Thr Glu Leu Lys Leu

595 600 605

Ser Asp Tyr Glu Gly Arg Leu Ile Gln Asn Ser Leu Thr Ile Glu Arg

610 615 620

Met Val Leu Ser Ala Phe Asp Glu Arg Arg Asn Lys Tyr Leu Glu Glu

625 630 635 640

His Pro Ser Ala Gly Lys Asp Pro Lys Lys Thr Gly Gly Pro Ile Tyr

645 650 655

Arg Arg Val Asp Gly Lys Trp Met Arg Glu Leu Val Leu Tyr Asp Lys

660 665 670

Glu Glu Ile Arg Arg Ile Trp Arg Gln Ala Asn Asn Gly Glu Asp Ala

675 680 685

Thr Ala Gly Leu Thr His Ile Met Ile Trp His Ser Asn Leu Asn Asp

690 695 700

Ala Thr Tyr Gln Arg Thr Arg Ala Leu Val Arg Thr Gly Met Asp Pro

705 710 715 720

Arg Met Cys Ser Leu Met Gln Gly Ser Thr Leu Pro Arg Arg Ser Gly

725 730 735

Ala Ala Gly Ala Ala Val Lys Gly Val Gly Thr Met Val Met Glu Leu

740 745 750

Ile Arg Met Ile Lys Arg Gly Ile Asn Asp Arg Asn Phe Trp Arg Gly

755 760 765

Glu Asn Gly Arg Lys Thr Arg Val Ala Tyr Glu Arg Met Cys Asn Ile

770 775 780

Leu Lys Gly Lys Phe Gln Thr Ala Ala Gln Arg Ala Met Met Asp Gln

785 790 795 800

Val Arg Glu Ser Arg Asn Pro Gly Asn Ala Glu Ile Glu Asp Leu Ile

805 810 815

Phe Leu Ala Arg Ser Ala Leu Ile Leu Arg Gly Ser Val Ala His Lys

820 825 830

Ser Cys Leu Pro Ala Cys Val Tyr Gly Pro Ala Val Ala Ser Gly Tyr

835 840 845

Asp Phe Glu Lys Glu Gly Tyr Ser Leu Val Gly Ile Asp Pro Phe Lys

850 855 860

Leu Leu Gln Asn Ser Gln Val Tyr Ser Leu Ile Arg Pro Asn Glu Asn

865 870 875 880

Pro Ala His Lys Ser Gln Leu Val Trp Met Ala Cys His Ser Ala Ala

885 890 895

Phe Glu Asp Leu Arg Val Ser Ser Phe Ile Arg Gly Thr Lys Val Ile

900 905 910

Pro Arg Gly Lys Leu Ser Thr Arg Gly Val Gln Ile Ala Ser Asn Glu

915 920 925

Asn Met Asp Thr Met Asp Ser Ser Thr Leu Glu Leu Arg Ser Arg Tyr

930 935 940

Trp Ala Ile Arg Thr Arg Ser Gly Gly Asn Thr Asn Gln Gln Arg Ala

945 950 955 960

Ser Ala Gly Gln Ile Ser Val Gln Pro Thr Phe Ser Val Gln Arg Asn

965 970 975

Leu Pro Phe Glu Lys Ser Thr Val Met Ala Ala Phe Thr Gly Asn Thr

980 985 990

Glu Gly Arg Thr Ser Asp Met Arg Ala Glu Ile Ile Arg Met Met Glu

995 1000 1005

Ser Ala Lys Pro Glu Glu Val Ser Phe Gln Gly Arg Gly Val Phe

1010 1015 1020

Glu Leu Ser Asp Glu Lys Ala Thr Asn Pro Ile Val Pro Ser Phe

1025 1030 1035

Asp Met Ser Asn Glu Gly Ser Tyr Phe Phe Gly Asp Asn Ala Glu

1040 1045 1050

Glu Tyr Asp Asn Gly Gly Gly Gly Ser Gly Gly Gly Gly Ser Gly

1055 1060 1065

Gly Gly Gly Ser Met Ser Leu Leu Thr Glu Val Glu Thr Pro Ile

1070 1075 1080

Arg Asn Glu Trp Gly Cys Arg Cys Asn Asp Ser Ser Asp Pro Leu

1085 1090 1095

Val Val Ala Ala Asn Ile Ile Gly Ile Leu His Leu Ile Leu Trp

1100 1105 1110

Ile Leu Asp Arg Leu Phe Phe Lys Cys Ile Tyr Arg Leu Phe Lys

1115 1120 1125

His Gly Leu Lys Arg Gly Pro Ser Thr Glu Gly Val Pro Glu Ser

1130 1135 1140

Met Arg Glu Glu Tyr Arg Lys Glu Gln Gln Asn Ala Val Asp Ala

1145 1150 1155

Asp Asp Ser His Phe Val Ser Ile Glu Leu Glu

1160 1165

**Immunogen #2. PB2NPM2 immunogen**

Met Asp Val Asn Pro Thr Leu Leu Phe Leu Lys Val Pro Ala Gln Asn

1 5 10 15

Ala Ile Ser Thr Thr Phe Pro Tyr Thr Gly Asp Pro Pro Tyr Ser His

20 25 30

Gly Thr Gly Thr Gly Tyr Thr Met Asp Thr Val Asn Arg Thr His Gln

35 40 45

Tyr Ser Glu Lys Gly Lys Trp Thr Thr Asn Thr Glu Thr Gly Ala Pro

50 55 60

Gln Leu Asn Pro Ile Asp Gly Pro Leu Pro Glu Asp Asn Glu Pro Ser

65 70 75 80

Gly Tyr Ala Gln His Phe Gln Arg Lys Arg Arg Val Arg Asp Asn Met

85 90 95

Thr Lys Lys Met Val Thr Gln Arg Thr Ile Gly Lys Lys Lys Gln Arg

100 105 110

Leu Asn Lys Arg Gly Tyr Leu Ile Arg Ala Leu Thr Leu Asn Thr Met

115 120 125

Thr Lys Asp Ala Glu Arg Gly Lys Leu Lys Arg Arg Ala Ile Ala Thr

130 135 140

Pro Gly Met Gln Ile Arg Gly Phe Val Tyr Phe Val Glu Thr Leu Ala

145 150 155 160

Arg Ser Ile Cys Glu Lys Leu Glu Gln Ser Gly Leu Pro Val Gly Gly

165 170 175

Asn Glu Lys Lys Ala Lys Leu Ala Asn Val Val Arg Lys Met Met Thr

180 185 190

Asn Ser Gln Asp Thr Glu Ile Ser Phe Thr Ile Thr Gly Asp Asn Thr

195 200 205

Lys Trp Asn Glu Asn Gln Asn Pro Arg Met Phe Leu Ala Met Ile Thr

210 215 220

Tyr Ile Thr Lys Asn Gln Pro Glu Trp Phe Arg Asn Ile Leu Ser Ile

225 230 235 240

Ala Pro Ile Met Phe Ser Asn Lys Met Ala Arg Leu Gly Lys Gly Tyr

245 250 255

Met Phe Glu Ser Lys Arg Met Lys Leu Arg Thr Gln Ile Pro Ala Glu

260 265 270

Met Leu Ala Ser Ile Asp Leu Lys Tyr Phe Asn Glu Ser Thr Lys Lys

275 280 285

Lys Ile Glu Lys Ile Arg Pro Leu Leu Ile Asp Gly Thr Ala Ser Leu

290 295 300

Ser Pro Gly Met Met Met Gly Met Phe Asn Met Leu Ser Thr Val Leu

305 310 315 320

Gly Val Ser Ile Leu Asn Leu Gly Gln Lys Lys Tyr Thr Lys Thr Thr

325 330 335

Tyr Trp Trp Asp Gly Leu Gln Ser Ser Asp Asp Phe Ala Leu Ile Val

340 345 350

Asn Ala Pro Asn His Glu Gly Ile Gln Ala Gly Val Asp Arg Phe Tyr

355 360 365

Arg Thr Cys Lys Leu Val Gly Ile Asn Met Ser Lys Lys Lys Ser Tyr

370 375 380

Ile Asn Lys Thr Gly Thr Phe Glu Phe Thr Ser Phe Phe Tyr Arg Tyr

385 390 395 400

Gly Phe Val Ala Asn Phe Ser Met Glu Leu Pro Ser Phe Gly Val Ser

405 410 415

Gly Val Asn Glu Ser Ala Asp Met Ser Ile Gly Val Thr Val Ile Lys

420 425 430

Asn Asn Met Ile Asn Asn Asp Leu Gly Pro Ala Thr Ala Gln Met Ala

435 440 445

Leu Gln Leu Phe Ile Lys Asp Tyr Arg Tyr Thr Tyr Arg Cys His Arg

450 455 460

Gly Asp Thr Gln Ile Gln Thr Arg Arg Ser Phe Glu Leu Lys Lys Leu

465 470 475 480

Trp Asp Gln Thr Gln Ser Lys Ala Gly Leu Leu Val Ser Asp Gly Gly

485 490 495

Pro Asn Leu Tyr Asn Ile Arg Asn Leu His Ile Pro Glu Val Cys Leu

500 505 510

Lys Trp Glu Leu Met Asp Glu Asp Tyr Arg Gly Arg Leu Cys Asn Pro

515 520 525

Leu Asn Pro Phe Val Ser His Lys Glu Ile Glu Ser Val Asn Asn Ala

530 535 540

Val Val Met Pro Ala His Gly Pro Ala Lys Ser Met Glu Tyr Asp Ala

545 550 555 560

Val Ala Thr Thr His Ser Trp Ile Pro Lys Arg Asn Arg Ser Ile Leu

565 570 575

Asn Thr Ser Gln Arg Gly Ile Leu Glu Asp Glu Gln Met Tyr Gln Lys

580 585 590

Cys Cys Asn Leu Phe Glu Lys Phe Phe Pro Ser Ser Ser Tyr Arg Arg

595 600 605

Pro Val Gly Ile Ser Ser Met Val Glu Ala Met Val Ser Arg Ala Arg

610 615 620

Ile Asp Ala Arg Ile Asp Phe Glu Ser Gly Arg Ile Lys Lys Glu Glu

625 630 635 640

Phe Ser Glu Ile Met Lys Ile Cys Ser Thr Ile Glu Glu Leu Arg Arg

645 650 655

Gln Lys Gly Gly Gly Gly Ser Gly Gly Gly Gly Ser Gly Gly Gly Gly

660 665 670

Ser Met Arg Arg Asn Tyr Phe Thr Ala Glu Val Ser His Cys Arg Ala

675 680 685

Thr Glu Tyr Ile Met Lys Gly Val Tyr Ile Asn Thr Ala Leu Leu Asn

690 695 700

Ala Ser Cys Ala Ala Met Asp Asp Phe Gln Leu Ile Pro Met Ile Ser

705 710 715 720

Lys Cys Arg Thr Lys Glu Gly Arg Arg Lys Thr Asn Leu Tyr Gly Phe

725 730 735

Ile Ile Lys Gly Arg Ser His Leu Arg Asn Asp Thr Asp Val Val Asn

740 745 750

Phe Val Ser Met Glu Phe Ser Leu Thr Asp Pro Arg Leu Glu Met Phe

755 760 765

Leu Tyr Val Arg Thr Asn Gly Thr Ser Lys Ile Lys Met Lys Trp Gly

770 775 780

Met Glu Met Arg Arg Cys Leu Leu Gln Ser Leu Gln Gln Ile Glu Ser

785 790 795 800

Met Ile Glu Ala Glu Ser Ser Val Lys Glu Lys Asp Met Thr Lys Glu

805 810 815

Phe Phe Glu Asn Lys Ser Glu Thr Trp Pro Ile Gly Glu Ser Pro Lys

820 825 830

Gly Val Glu Glu Gly Ser Ile Gly Lys Val Cys Arg Thr Leu Leu Ala

835 840 845

Lys Ser Val Phe Asn Phe Asp Leu Gly Gly Leu Tyr Glu Ala Ile Glu

850 855 860

Glu Cys Leu Ile Asn Asp Pro Trp Val Leu Leu Asn Ala Ser Trp Phe

865 870 875 880

Asn Ser Phe Leu Thr His Ala Leu Lys Gly Ser Gly Gly Ser Gly Met

885 890 895

Ser Leu Leu Thr Glu Val Glu Thr Tyr Val Leu Ser Ile Val Pro Ser

900 905 910

Gly Pro Leu Lys Ala Glu Ile Ala Gln Arg Leu Glu Asp Val Phe Ala

915 920 925

Gly Lys Asn Thr Asp Leu Glu Ala Leu Met Glu Trp Leu Lys Thr Arg

930 935 940

Pro Ile Leu Ser Pro Leu Thr Lys Gly Ile Leu Gly Phe Val Phe Thr

945 950 955 960

Leu Thr Val Pro Ser Glu Arg Gly Leu Gln Arg Arg Arg Phe Val Gln

965 970 975

Asn Ala Leu Asn Gly Asn Gly Asp Pro Asn Asn Met Asp Arg Ala Val

980 985 990

Lys Leu Tyr Arg Lys Leu Lys Arg Glu Ile Thr Phe His Gly Ala Lys

995 1000 1005

Glu Ile Ala Leu Ser Tyr Ser Ala Gly Ala Leu Ala Ser Cys Met

1010 1015 1020

Gly Leu Ile Tyr Asn Arg Met Gly Ala Val Thr Thr Glu Val Ala

1025 1030 1035

Phe Gly Leu Val Cys Ala Thr Cys Glu Gln Ile Ala Asp Ser Gln

1040 1045 1050

His Arg Ser His Arg Gln Met Val Thr Thr Thr Asn Pro Leu Ile

1055 1060 1065

Arg His Glu Asn Arg Met Val Leu Ala Ser Thr Thr Ala Lys Ala

1070 1075 1080

Met Glu Gln Met Ala Gly Ser Ser Glu Gln Ala Ala Glu Ala Met

1085 1090 1095

Glu Val Ala Ser Gln Ala Arg Gln Met Val Gln Ala Met Arg Ala

1100 1105 1110

Ile Gly Thr His Pro Ser Ser Ser Thr Gly Leu Lys Asp Asp Leu

1115 1120 1125

Leu Glu Asn Leu Gln Ala Tyr Gln Lys Arg Met Gly Val Gln Met

1130 1135 1140

Gln Arg Phe Lys

1145
